# Role of *VEGFA* gene polymorphisms in colorectal cancer patients who treated with bevacizumab

**DOI:** 10.18632/oncotarget.22295

**Published:** 2017-11-06

**Authors:** Wei Cui, Feng Li, Qiang Yuan, Gang Chen, Cailing Chen, Bo Yu

**Affiliations:** ^1^ Department of General Surgery, The Military General Hospital of Beijing PLA, Beijing 100700, China; ^2^ Department of Health, The Military General Hospital of Beijing PLA, Beijing 100700, China

**Keywords:** VEGFA, polymorphisms, bevacizumab, colorectal cancer

## Abstract

**Objectives:**

This study aimed to explore the effects of vascular endothelial growth factor A (*VEGFA*) gene polymorphisms (rs699947 and rs833061) on Bevacizumab (BEV) treatment in colorectal cancer (CRC) patients.

**Methods:**

125 CRC cases receiving BEV plus FOLFIRI treatment were recruited in this study. *VEGFA* polymorphisms were genotyped using polymerase chain reaction-restriction fragment length polymorphism (PCR-RFLP) method. Correlation of *VEGFA* gene polymorphisms with the response rate and progression free survival (PFS) was evaluated. Multivariate analyses were performed to estimate the effects of *VEGFA* polymorphisms on the therapeutic effects of BEV treatment in CRC patients.

**Results:**

Rs699947 variants did not show significant association with BEV treatment. For rs833061 analysis, TT and TC genotype carriers had significantly higher ORR (objective response rate) than CC carriers (*P*=0.048 and *P*=0.021, respectively). Moreover, TT carriers underwent a well DCR (disease control rate) compared to CC carriers (*P*=0.002). PFS time also showed obvious correlation with rs833061 polymorphism (log rank test, *P*=0.002). Multivariate analyses demonstrated that TT and TC genotypes of rs833061 polymorphism were significantly correlated with enhanced therapeutic effects and prolonged PFS in CRC patients.

**Conclusion:**

*VEGFA* rs833061 polymorphism is significantly associated with the therapeutic efficiency of bevacizumab in CRC patients.

## INTRODUCTION

Colorectal cancer (CRC) is one of the most common malignancies, which is caused by the abnormal growth of cells in colon or rectum part. The tumor cell could spread elsewhere in the body. Recent years, with the development of therapy method, the mortality rate of CRC exhibits decreased trend [1]. CRC is caused by various genetic and environmental factors [2–5]. In the tumor growth and metastasis, angiogenesis is an essential factor [6]. Vascular endothelial growth factor (VEGF), a super family of growth factors, is involved in both vasculogenesis and angiogenesis [7, 8]. It has been reported that the inhibition of angiogenesis will contribute to anti-tumor treatments [9, 10]. Recent decades, many angiogenesis inhibitors have been applied for cancer therapy. Bevacizumab (BEV) is a humanized monoclonal antibody which could suppress the angiogenesis by inhibiting VEGF binding to the VEGF receptors (VEGFR). Therefore, BEV regulates the angiogenesis via the inhibition of signal transduction. BEV has been used in the treatment of metastatic CRC. However, there was few clinical or biological factors to predict the therapeutic efficiency of BEV.

VEGFA is an important regulator of angiogensis in VEGF family, represents the main target of BEV. VEGFA plays a potential role in the development and treatment of CRC. Previous studies showed that polymorphisms of the *VEGFA* gene influenced the expression or the function of its coded protein [11–13]. Therefore, the polymorphisms may have impact on the onset and progression of tumors [14, 15]. It has been reported that single nucleotide polymorphisms (SNPs) of *VEGFA* gene could affect the tumor susceptibility, severity, and/or survival [16–18].

The role of *VEGFA* SNPs on the therapeutic efficiency of BEV-based patients has been investigated in many tumors [18–21]. For examples, Sohn et al. reported that TT genotype of *VEGFA* rs833061 polymorphism predicted superior ORR (objective response rate) to the first-line cytotoxic chemotherapy combined with bevacizumab among CRC patients in South Korea [21]. The GeparQuinto Phase III trial (NCT00567554) demonstrated that *VEGFA* gene rs699947 SNP was strongly correlated with pathological complete response among breast cancer patients receiving BEV-based neoadjuvant chemotherapy [22]. Based on the related investigations, we speculated that *VEGFA* gene rs699947 and rs833061 polymorphisms might influence treatment response among CRC patients treated by BEV-based chemotherapy. However, there were merely no similar researches on the issue among Chinese Han CRC patients. Minor allele frequencies (MAF) of *VEGFA* gene rs699947 (c.-2578A>C) and rs833061 (c.-1498C>T) polymorphisms are more than 0.05 in CHB (Chinese Han in Beijing) population. Therefore, we investigated the predictive function of *VEGFA* gene rs699947 and rs833061 polymorphisms in the outcomes of BEV-based therapy in Chinese Han CRC patients.

## RESULTS

### Baseline characteristics

A total of 125 (mean age 62.50±8.45 years) CRC patients receiving BEV combined FOLFIRI treatment were collected in this study Baseline characteristics of the participants were summarized in Table 1.

**Table 1:** Baseline characteristics

Characteristics	Case number
**Age**	
≤60	44(35.20)
>60	81(64.8)
**Gender**	
Male	81(64.80)
Female	44(35.20)
**Primary tumor site**	
Right colon	33(26.40)
Left colon	55(44.00)
Rectum	37(29.60)
**ECOG PS**	
0	96(76.80)
1-2	29(23.20)
**Primary tumor resected**	
No	94(75.20)
Yes	31(24.80)
**Metastatic number**	
1	58(46.40)
>1	67(53.60)

### Treatment response

After three months treatment, 5 patients reached CR, 61 cases reached PR, 38 patients reached SD, and 21 cases presented PD. The ORR was 52.8%, and DCR was 83.2%. Meanwhile, the mean PFS were 19.67 ± 0.919 months during 30-month follow up investigation.

### Genotype distributions of *VEGFA* rs699947 and rs833061 polymorphisms

In the current study, PCR-RFLP was performed to identify the genotype of *VEGFA* rs699947 and rs833061 polymorphisms. In addition, the results of 50 random samples were verified by direct sequencing. The genotype confirmed by PCR-RFLP and direct sequencing technology exhibited similar, suggesting the high accuracy of PCR-RFLP method in genotyping.

Genotypes and alleles distributions of rs699947 and rs833061 polymorphisms did not deviate from HWE test, showing the good representativeness of the objects. The genotype distributions of rs699947 and rs833061 among the study population were summarized in Table 2.

**Table 2:** Association between *VEGFA* gene polymorphisms and therapeutic effects of bevacizumab among CRC patients

SNPs	Total cases	ORR		DCR		PFS	
		Case number	*P*	Case number	*P*	Mean time (months)	*P^a^*
rs699947							
CC	74 (59.20)	35 (58.33)	0.752	66 (63.46)	0.057	19.68 ± 1.11	log rank test, *P*=0.392
CA	46 (36.80)	29 (48.33)	0.316	35 (33.65)	0.433	20.98 ± 1.73	
AA	5 (4.00)	2 (3.33)	Reference	3 (2.88)	Reference	16.60 ± 4.63	
rs833061							
TT	79 (63.20)	42 (63.64)	0.048	70 (67.31)	0.002	20.41 ± 1.20	log rank test, *P*=0.002*
TC	36 (28.80)	22 (33.33)	0.021	29 (27.88)	0.052	17.81 ± 1.29	
CC	10 (8.00)	2 (3.03)	Reference	5 (4.81)	Reference	11.82 ± 2.29	

Note: ^a^: significant level was adjusted by Bonferroni method, α=0.017.

### Effects of rs699947 and rs833061 SNPs on BEV therapeutic efficiency

**Figure 1:**
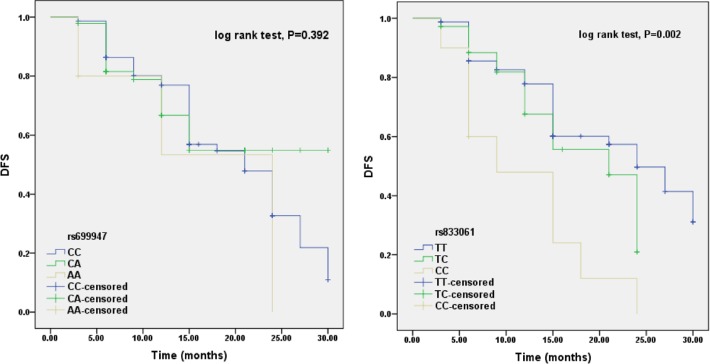
Effects of rs699947 and rs833061 polymorphisms on PFS among CRC patients receiving BEV treatments There was no significant association between rs699947 polymorphism with PFC (log rank test, *P*=0.392). While rs833061 polymorphism showed obvious correlation with PFS among the study population (log rank test, *P*=0.002). TT carriers had a significantly better PFS than CC carriers (log rank test, *P*=0.001), while patients carrying TC genotype had obviously better PFS than those carrying CC genotype (log rank test, *P*=0.013). There was no remarkable difference between TT carriers and TC carriers (log rank test, *P*=0.126). The significant level was adjusted by Bonferroni method, α=0.017.

In order to estimate the effects of rs699947 and rs833061 SNPs on BEV therapeutic efficiency, we compared ORR, DCR and PFS based on the genotypes of rs699947 and rs833061 polymorphisms (Table 2, Figure 1). In this study, there were no significant association observed between rs699947 polymorphism and ORR, DCR or PFS (*P*>0.05 for all, Table 2). For rs833061 analysis, TT and TC genotype carriers had significantly higher ORR than CC carriers (*P*=0.048 and *P*=0.021, respectively). Moreover, TT carriers underwent a well DCR compared to CC carriers (*P*=0.002). The PFS time also showed obvious correlation with rs833061 polymorphism (log rank test, *P*=0.002; significant level was adjusted by Bonferroni method, α=0.017). TT carriers had a significantly better PFS than CC carriers (log rank test, *P*=0.001), while patients carrying TC genotype had obviously better PFS than those carrying CC genotype (log rank test, *P*=0.013). There was no remarkable difference between TT carriers and TC carriers (log rank test, *P*=0.126) (Figure 1).

After adjusted for age and gender, rs833061 TC carriers exhibited significantly higher ORR than CC carriers (OR=0.159, 95%CI=0.029-0.871, *P*=0.034), while TT carriers had a better DCR (OR=0.129, 95%CI=0.031-0.532, *P*=0.002) (Table 3). Univariate cox analysis demonstrated that TT and TC genotypes were protective factors for PFS (*P*=0.002 and *P*=0.048, respectively). After adjusting to age and gender, TT (HR=0.299, 95%CI=0.141-0.634, *P*=0.002) and TC (HR=0.449, 95%CI=0.203-0.991, *P*=0.048) genotypes also showed obvious correlation with PFS (Table 3).

**Table 3:** The effects of VEGFA rs833061 polymorphism on ORR, DCR and PFS among CRC patients receiving bevacizumab treatment

Genotypes	ORR				DCR				PFS			
	OR	*P*	OR^a^	*P*^a^	OR	*P*	OR^a^	*P*^a^	HR	*P*	HR^b^	*P*^b^
TT	0.220 (0.044-1.103)	0.048	0.224 (0.044-1.134)	0.071	0.129 (0.031-0.532)	0.002	0.124 (0.030-0.519)	0.004	0.299 (90.141-0.634)	0.002	0.299 (0.141-0.634)	0.002
TC	0.159 (0.029-0.861)	0.021	0.159 (0.029-0.871)	0.034	0.241 (0.054-1.070)	0.052	0.230 (0.051-1.038)	0.056	0.449 (0.203-0.991)	0.048	0.449 (0.203-0.991)	0.048
CC	Reference				Reference				Reference			

Notes: a: calculated by logistic regression analysis; b: analyzed by cox-regression analysis.

## DISCUSSION

Accumulation of VEGF plays promoting roles in the development of tumors via enhancing angiogenesis. Among the VEGF family, VEGFA has the strongest activity to promote the angiogenesis. BEV is aimed at VEGF, weakens or blocks the interaction between VEGF and VEGFR, thus inhibits the proliferation of endothelial cell, reduces the angiogenesis in tumorigenesis. Inhibition of angiogenesis could prevent the growth of tumors and increase the therapeutic efficacy of radiotherapy and chemotherapy [23]. As one of the most common malignancy in large intestine, CRC has a high incidence and mortality. Recent years, relevant researches showed that therapeutic efficacy of BEV monotherapy is very low [24], but the combination of chemotherapy and BEV significantly improve the therapeutic efficacy of advanced CRC [25]. However, the therapy response is different in the participants who receive the same therapy method. Additionally, the toxic and adverse effects are also different in the patients. These phenomena may be decided by the individual difference.

*VEGFA*, one of the target gene of BEV, is located at 6p12, and contains 9 exons. It has been reported that polymorphisms in *VEGFA* gene play a potential role in the development of many tumors, including CRC [14, 15, 26]. Polymorphisms in this gene may impact the therapeutic efficiency of BEV. Ferroni et al demonstrated that −1154AA genotype was negatively correlated with efficacy and toxicity of bevacizumab in metastatic cancer patients [18]. Di and colleagues suggested that rs2010963 genotype was correlated with longer PFS in recurrent glioblastomas patients for the response to bevacizumab [20]. Rs699947 (c.-2578A>C) and rs833061 (c.-1498C>T) are the two widely researched SNPs in the promoter region of *VEGFA* gene. Previous studies showed that these polymorphisms could affect the promoter activity of the *VEGFA* gene, and then alter the production of VEGFA [12, 13]. As a target of BEV treatment, abnormal accumulation of VAGFA might contribute to angiogenesis, thus leading to therapeutic failures. It was reported that high level of VEGFA predicted shorter recurrence-free survival among patients receiving bevacizumab-based chemotherapy [27]. Thus, polymorphisms at promoter region of VAGFA gene may significantly influence the therapeutic efficacy of BEV chemotherapy. Because the BEV monotherapy has low therapeutic efficacy, we used the combination chemotherapy to explore the effects of *VEGFA* polymorphisms in the BEV therapeutic efficacy for CRC.

Growing evidences have suggested that BEV could significantly enhance the PFS of CRC patients who are treated with combination chemotherapy [25]. However, the therapeutic efficacy of BEV may exhibit individual differences. Genetic factors play pivotal roles in individual differences. To explore the association between SNPs and BEV treatments can help the physicians to identify the population who will benefit from BEV combined chemotherapy among advanced CRC patients, thus optimizing the management of BEV. In this study, we explored the association of *VEGFA* polymorphisms with therapeutic efficacy of BEV in CRC patients. The results were estimated by ORR, DCR, and PFS. Analysis results indicated that rs699947 polymorphism did not show significant association with BEV treatment in CRC patients. Additionally, we found that the genotypes of the rs833061 SNP were significantly correlated with ORR, DCR and PFS among the study population. Multivariate analysis indicated that TC carriers had a higher ORR, while TT carriers underwent well DCR. Furthermore, both of TC and TT carriers had a significantly better PFS than CC carriers. That was partly accordance with previous studies. Pallaud et al. found that among non-squamous non-small-cell lung cancer patients receiving BEV plus chemotherapy, TT carriers of rs833061 had the highest response rate [28]. Loupakis et al. suggested that rs699947 variant could not impact the PFS and OS in CRC patients in Caucasian population [29]. Schneider et al. suggested that rs833061 TT might decrease the hypertension toxicity of BEV [30]. *VEGF* −2578CC and −1498TT genotypes decreased the hypertension toxicity of BEV in Japanese CRC patients [31], but these two SNPs had no association with the outcome of CRC patients treated with BEV-based chemotherapy [32]. However, other studies were different from our results. For instance, Loupakis et al. revealed that rs833061 TT genotype associated with shorter PFS in Italian CRC patients who received first-line FOLFIRI plus BEV treatment [29]. The divergence may be caused by the diversity of regions, races, select criteria or the categories of disease.

In conclusion, *VEGFA* rs833061 polymorphism is obviously associated with the therapeutic efficiency of BEV in Chinese Han CRC patients. This result will help us to select the patients who exert high response to BEV treatment, thus improving the clinical outcomes of CRC patients. However, our results may be limited by the relatively small sample size and the single ethnicity in the current study. Additionally, the concrete molecular mechanisms underlying the genetic association of *VEGFA* rs833061 polymorphism with BEV treatment are not investigated in the current study. Further investigations will be required to verify the conclusion.

## MATERIALS AND METHODS

### Study subjects

This study was approved by the local ethics committee. All of the participants were unrelated Chinese Han people. They understood the objectives of the study and signed the informed consents. The sample collection was in accordance with the Helsinki declaration.

Study subjects were selected from CRC patients who were hospitalized in The Military General Hospital of Beijing PLA from January 2010 to January 2013. Patients were diagnosed with CRC by computed tomography (CT) and histopathologic examinations based on the diagnosis standards of CRC [33]. Include criteria were as follows: CRC patients who had metastasis or postoperative recurrence were included in this study. Patients had the following conditions were excluded from this study: expectancy life was less than 3 months; received anti-angiogenic therapy before this study; and had any other cardiovascular or cerebrovascular diseases. Patients who had the adjuvant treatment less than 6 cycles were also excluded from this study. Finally, 125 patients (age range 31-80 years) were recruited in this study.

### Therapy methods and therapeutic efficacy

All the patients received BEV (5 mg/kg, every 2 weeks) plus FOLFIRI treatment. FOLFIRI therapeutic regimen included Irinotecan 180 mg/m^2^, l-leucovorin 200 mg/m^2^, and 5-fluorouracil 400 mg/m^2^, intravenous injection on day 1, followed by a 4-6hrs infusion of 5-fluorouracil 2,400 mg/m^2^, every 2 weeks.

Treatment response was evaluated by experienced oncologists according to RECIST 1.1 after three months treatment [34]. Treatment response included complete response (CR), partial response (PR), stable disease (SD) and progressive disease (PD). The short therapeutic efficiency was estimated by object response rate (ORR) and disease control rate (DCR). The rate of CR+PR is defined as ORR. DCR is defined as the rate of CR+PR+SD. The long effects of VEGFA variants on BEV treatments were evaluated by progression-free survival (PFS). PFS is the duration from the beginning of the treatment to the first observation of disease progression or the last follow-up visit. Follow up time was 30 months. Patients were diagnosed by CT scan every 3 months.

### Genotyping analysis

5 ml peripheral venous blood was collected form each participant who had a 12h fasting before the adjuvant treatment. Blood samples were anti-coagulated by EDTA, and stored at −80°C until used. Genomic DNA was extracted by a TIANamp Blood DNA Kit (Tiangen, China), following the manufacturer's protocol.

SNPs of *VEGF* gene were analyzed by polymerase chain reaction-restriction fragment length polymorphism (PCR-RFLP), according to the previous study [35]. Genotype results were confirmed by direct sequencing method using ABI 3730XL sequencer via randomly selected 50 samples.

### Statistical analysis

All of the statistic analyses were conducted by PASW 18.0 software. Significant level was set to two side 0.05 and adjusted by Bonferroni method in multiple comparison. Genotype and allele distributions were detected by Hardy-Weinberg equilibrium (HWE) test. Genotype and allele frequencies were obtained by direct counting. Quantitative variables were shown as mean± standard deviation. Qualitative variables were analyzed by Chi-square test. CR, PR, SD and PD were used to present the responses for bevacizumab treatment. Kaplan-Meier survival curve was utilized to analyze the PFS of the patients after bevacizumab therapy. Time-to-event distributions were analyzed by log-rank test, and the results were adjusted using Bonferroni method. Multivariate logistic regression analysis and multivariate cox regression analysis were performed to estimate the association of *VEGF* polymorphisms on bevacizumab efficiency after adjusting to the other confusing factors.
